# Primary scene responses by Helicopter Emergency Medical Services in New South Wales Australia 2008–2009

**DOI:** 10.1186/1472-6963-12-402

**Published:** 2012-11-15

**Authors:** Colman B Taylor, Bette Liu, Eleanor Bruce, Brian Burns, Stephen Jan, John Myburgh

**Affiliations:** 1 The George Institute for Global Health, PO Box M201, Missenden Rd Camperdown, NSW 2050, Sydney, NSW, Australia; 2 The University of Sydney, Sydney Medical School, Sydney, NSW, Australia; 3 The University of NSW, Faculty of Medicine, Sydney, NSW, Australia; 4 The University of Sydney, School of Geosciences, Sydney, NSW, Australia; 5 Greater Sydney Area HEMS, Ambulance Service of NSW, Sydney, NSW, Australia

**Keywords:** Wounds and injury, Trauma systems, Helicopter Emergency Medical Services, Patient acuity, Cost, Reimbursement

## Abstract

**Background:**

Despite numerous studies evaluating the benefits of Helicopter Emergency Medical Services (HEMS) in primary scene responses, little information exists on the scope of HEMS activities in Australia. We describe HEMS primary scene responses with respect to the time taken, the distances travelled relative to the closest designated trauma hospital and the receiving hospital; as well as the clinical characteristics of patients attended.

**Methods:**

Clinical service data were retrospectively obtained from three HEMS in New South Wales between July 2008 and June 2009. All available primary scene response data were extracted and examined. Geographic Information System (GIS) based network analysis was used to estimate hypothetical ground transport distances from the locality of each primary scene response to firstly the closest designated trauma hospital and secondly the receiving hospital. Predictors of bypassing the closest designated trauma hospital were analysed using logistic regression.

**Results:**

Analyses included 596 primary missions. Overall the HEMS had a median return trip time of 94min including a median of 9min for activation, 34min travelling to the scene, 30min on-scene and 25min transporting patients to the receiving hospital. 72% of missions were within 100km of the receiving hospital and 87% of missions were in areas classified as ‘major cities’ or ‘inner regional’. The majority of incidents attended by HEMS were trauma-related, with road trauma the predominant cause (44%). The majority of trauma patients (81%) had normal physiology at HEMS arrival (RTS = 7.84). We found 62% of missions bypassed the closest designated trauma hospital. Multivariate predictors of bypass included: age; presence of spinal or burns trauma; the level of the closest designated trauma hospital; the transporting HEMS.

**Conclusion:**

Our results document the large distances travelled by HEMS in NSW, especially in rural areas. The high proportion of HEMS missions that bypass the closest designated trauma hospital is a seldom mentioned benefit of HEMS transport. These results along with the characteristics of patients attended and the time HEMS take to complete primary scene responses are useful in understanding the benefit HEMS provides and the services it replaces.

## Background

In New South Wales (NSW) Australia, Helicopter Emergency Medical Services (HEMS) undertake primary scene responses and secondary inter-facility transfers as part of the state trauma plan and critical care networks [1,2].

Compared to road transport systems, there are three predominant advantages of using HEMS for a primary scene response. They include faster transport to definitive care, access to patients where limited infrastructure precludes timely road access, and direct delivery to the patient of advanced life-saving critical skills by a specialty trained physician or paramedic. During patient transport, a further advantage of HEMS is the ability to bypass regional hospitals and transfer patients directly to hospitals which have appropriate facilities, as per local trauma treatment guidelines [2]. In NSW, a HEMS primary response is recommended in scenarios including difficult patient access (e.g. cliff fall or water rescue), where the patient condition requires specialised interventions (e.g. rapid sequence intubation) or situations where a helicopter will provide a more expedient response and transport [3,4]. In spite of its high costs, recent evidence indicates that HEMS are potentially cost-effective [5] but this is dependent on the accuracy of triage [6].

To understand the role of HEMS as a distinct intervention in primary scene responses, it is necessary to evaluate the effect of HEMS on patient outcome, compared to conventional ground transport [7-11]. However there are many challenges to such studies as HEMS encompass many aspects such as decreasing the time to definitive care (logistics), and rapidly providing potentially life-saving critical interventions. Both within and between jurisdictions, HEMS are also known to vary in aspects such as staffing and skill levels, range of operations (e.g. pre-hospital care, inter-hospital retrieval and SAR) and the types of patients attended. Therefore, to understand how a HEMS may benefit patients from primary scene responses in NSW, a fundamental step is to provide an understanding of HEMS operations with respect to the key time performance indicators, the proximity of operations to the destination hospital and the types of the patients attended. Further, although HEMS are known to travel large distances, the proportion of missions which bypass closer designated trauma hospitals has not been previously estimated in NSW.

### Aim

The aim of this study was to document the scope of HEMS primary scene responses in NSW with respect to the time taken, the distances travelled and the clinical characteristics of patients attended. Additionally, this study examines the proportion of missions which bypass the closest designated trauma hospital and the predictors of hospital bypass.

## Methods

### Setting

The state of NSW is situated on the east coast of Australia and is characterized by a large land mass (over 800,000 Square Km) and a population of approximately 6.8 million people, who predominantly reside near coastal areas. The capital city is Sydney which incorporates approximately two thirds of the population of NSW (approximately 4.5 million). As of the 1st July 2008, the NSW trauma care system incorporated a networked system of 23 designated trauma hospitals, which were classified as either major adult (n=9), major paediatric (n=3), regional (n=2) or rural regional (n=10) according to available resources [2]. During this time, nine HEMS operated in NSW, performing primary scene responses and secondary inter-facility transfers as part of the NSW trauma system. HEMS are activated by service protocols according to MIST criteria (Mechanism of injury; Injuries sustained; physiological Signs and symptoms; Transport time) or via a rapid launch coordinator. Three HEMS are located in the Sydney metropolitan and the remaining six HEMS are located in regional areas of NSW [12]. One of the metropolitan services operated a separate rapid response trauma-only service as part of an ongoing clinical trial [13].

### Data collection

This study was approved by the Sydney South West Area Health Service HREC. We performed a retrospective cross-sectional analysis of clinical service data for primary scene responses collected by three HEMS (Greater Sydney Area HEMS) in NSW for the period 1^st^ July 2008 through 30^th^ June 2009 operated by the Ambulance Service of NSW.

Clinical service and key timing data are collected by each HEMS service in NSW for each patient attended to by a HEMS crew. Data are usually recorded by the medical crew during the mission and then transcribed contemporaneously onto case sheets. These are entered into a database following the mission. Data fields include date of transport; components of transport time (e.g. time trip is activated; time arrived at patient); mission type (primary scene response or secondary inter-facility transport); patient diagnosis categories; transport origin and destination; and clinical interventions undertaken by HEMS staff. Patient clinical observation data are also collected from time of patient contact until stretcher offload on completion of the mission.

Of the nine HEMS operating in NSW during the reference period, consistent clinical service data for primary scene responses were available in four HEMS only. This included two HEMS based in the Sydney metropolitan area (Metro1 & Metro2) and two HEMS located in regional population centres (Regional1 and Regional2). Three HEMS were operated by the same provider (Greater Sydney Area HEMS). One of the metropolitan HEMS (Metro2) with consistent data was operated by Careflight Ltd. During the study period, the CareFlight HEMS was solely operating within the confines of a head injury trial (HIRT) and was therefore excluded.

### Hypothetical ground transport distance estimation and remoteness classification

For each HEMS primary scene response we estimated a corresponding hypothetical ground transport distance, using a Geographic Information Systems (GIS) based network analysis. Response locations were mapped using Google Earth and imported into the GIS. A road network layer was compiled using GEODATA TOPO 250k Map Series (Geoscience Australia) to model vehicle transport routes. The ArcGIS 9.3.1 Network Analysis extension was used to model the travel distance between each incident location and firstly the nearest designated trauma hospital and secondly the receiving trauma hospital based on the optimal travel route. In identifying optimal travel routes, travel impedance factors (e.g. gravel roads) were established to account for variability in travel speed associated with the road type.

To assess the ‘remoteness’ of the populations serviced by each HEMS we used the enhanced Accessibility/Remoteness Index of Australia (ARIA+) score [14]. The ARIA+ scores localities according to their proximity to service centres using a continuous scale from 0 (high accessibility) to 15 (high remoteness). Scores can be further classified into 5 major categories: major cities, inner regional, outer regional, remote and very remote. We recorded the proportion of responses in each ARIA+ category according to the postcodes attended by each HEMS during the 2008/2009 financial year.

### Data analysis

Data were analysed using SAS version 9.2. For the analysis of HEMS time and distance we included only primary scene responses categorised as ‘emergency’ as these correspond to time critical missions that require a rapid response. We also excluded missions requiring a winch as we assumed these missions can only be completed by HEMS (and ground distance was unable to be calculated). For each HEMS primary scene response the following times were calculated: activation time (time between activation call received and helicopter departure from base to the scene), response time (time between departure from base to arrival at scene), scene time (time between arriving at the patient and departing the scene for destination hospital), transport time (time between departing the scene and arriving at destination hospital) and total time (time between activation call received and arriving at destination hospital).

We described patient demographics, clinical characteristics and diagnostic groups for all primary scene responses. We defined trauma patients according to the incident recorded in the service database, such as ‘motor vehicle accident’ or ‘fall’. For trauma patients, we described the types of trauma sustained (such as chest or head trauma) according to the APACHE III sub-diagnosis recorded in the HEMS database [15]. For non-trauma patients, diagnoses were described according to the APACHE III titles (such as cardiovascular or metabolic).

Continuous variables including age, Glasgow Coma Score (GCS) [16] and Revised Trauma Score (RTS) [17] were categorised according to standard definitions. The association between potential predictors such as type of trauma sustained and whether or not patients were taken to the closest designated trauma hospital were analysed using stepwise logistic regression. Each potential predictor was firstly examined in a univariate model and factors with a predetermined level of significance (p<0.1) were then entered into a multivariate model.

## Results

During the reference period, a total of 596 primary scene responses were identified from clinical service data in the three HEMS. The metropolitan HEMS undertook more missions (N=411, 69%) than the Regional HEMS (Regional1 N=102, 17%; Regional2 N=83, 14%). After excluding non-emergency missions and winch missions, 464 primary scene responses were used to calculate distance and time (78% of total missions [Metro1 HEMS N=308, 66%; Regional1 HEMS N=74, 16%; Regional2 HEMS N=82, 18%]).

Table 1 shows the mean and median time taken for the HEMS non-winch and emergency responses (N=464). Overall, a return trip took a median time of 94min. Based on the inter-quartile range of each time component, between 5%-11% of time was spent in activation, between 27%-48% was spent travelling to the scene, between 21%-43% was spent on-scene and between 27%-37% was spent transporting the patient to the receiving hospital.

**Table 1:** Time categories for HEMS ‘emergency’ and non-winch primary scene responses

**Time Category**	**N**	**Median min (IQR)**	**Mean min (SD)**
Activation	464	9 (5–10)	8.7 (6.4)
Response	464	34 (25–45)	38.7 (21.4)
Scene	464	30 (20–40)	34.7 (22.3)
Transport	450	25 (19–35)	29.6 (23.4)
Total	450	94 (75–115)	102.4 (48.1)

**Figure 1:**
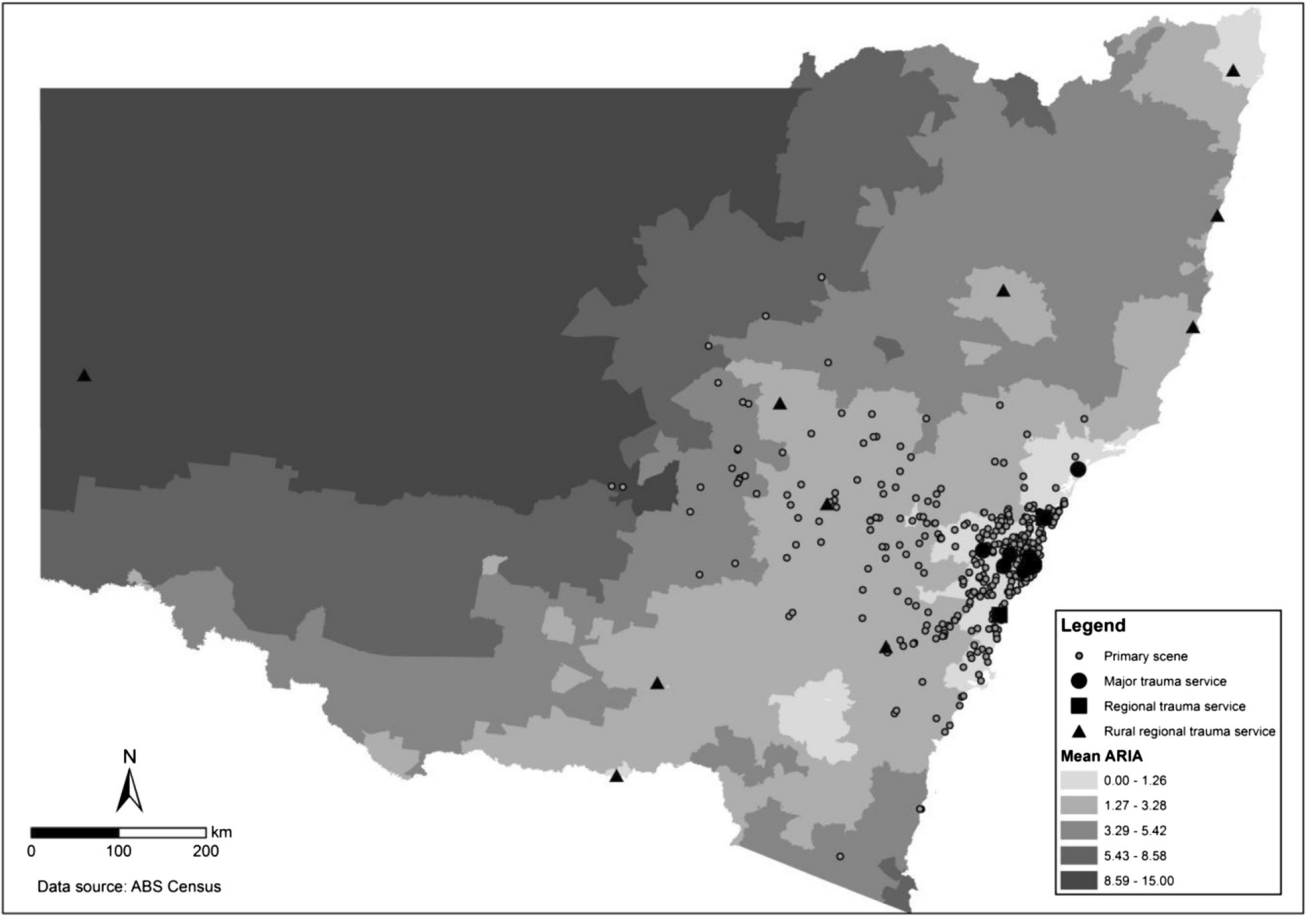
Map of NSW including remoteness index (ARIA+), trauma services and location of primary scene responses during July 2008 to June 2009.

Our data allowed us to estimate the road distance from the response location to the nearest designated trauma hospital and the actual receiving hospital for 425 (92%) and 406 (88%) primary scene responses respectively (Figure 1). For the 406 missions in which we had information on the receiving hospital, we found HEMS bypassed the closest designated trauma hospital in 62% of cases (N = 406).

Table 2 shows the patient and HEMS characteristics that were associated with bypassing the closest designated trauma hospital. In univariate analyses we found classification of spinal or burns trauma, along with age, GCS, the level of closest designated trauma hospital and the service all met the pre-defined level of significance (P<0.1) and were therefore included in a multivariate model. After adjusting for all factors in the multivariate analysis, results showed patients with spinal or burns trauma were less likely to be taken to the closest hospital compared to patients without both these types of trauma (OR: 0.47 p=0.055 [spinal]; OR: 0.13 p=0.046 [burns]). Regarding age, results showed paediatric patients (<=16 years) were less likely to be taken to the closest hospital compared to adult patients (OR: 0.48; p=0.042). We also found the level of closest designated trauma hospital was a highly significant predictor of bypass (p<0.0001) with patients less likely to be taken to the closest hospital if it was classified as regional (OR: 0.16) and rural regional (OR 0.09) compared to hospitals classified as major trauma. Finally, the individual HEMS transporting the patient also predicted hospital bypass with both Regional1 and Regional2 more likely to take patients to the closest designated trauma hospital (OR: 3.61 & 10.95; p<0.0001 & 0.001 respectively) compared to Metro1.

**Table 2:** Univariate and multivariate predictors of HEMS transports being taken to the closest designated trauma hospital

**Predictor**	**Category**	**N closest/total**	**%**	**Univariate OR (95% CI)**	**Multivariate OR (95% CI)**	**Multivariate p-value**
CHEST TRAUMA	YES	22/55	40.0%	1.09 (0.61 - 1.95)		
	NO	133/351	37.9%	1.00		
HEAD TRAUMA	YES	50/143	35.0%	0.81 (0.53 - 1.24)		
	NO	105/263	39.9%	1.00		
SPINAL TRAUMA	YES	22/80	27.5%	0.55 (0.32 - 0.94)	0.47 (0.22 - 0.99)	p=0.055
	NO	133/326	40.8%	1.00	1.00	
BURNS	YES	1/10	10.0%	0.18 (0.02 - 1.39)	0.13 (0.02 - 1.05)	p=0.046
	NO	154/396	38.9%	1.00	1.00	
AGE^1^	<=16	18/64	28.1%	0.58 (0.33 - 1.05)	0.48 (0.24 - 0.97)	p=0.042
	17+	136/339	40.1%	1.00	1.00	
GCS^2^	3-8	9/39	23.1%	0.46 (0.21 - 0.99)	0.57 (0.23 - 1.4)	p=0.219
	9-15	143/361	39.6%	1.00	1.00	
RTS^3^	< 7.84	18/64	28.1%	0.75 (0.44 - 1.27)		
	7.84	115/294	39.1%	1.00		
CLOSEST HOSPITAL	MAJOR	102/208	49.0%	1.00	1.00	
	REGIONAL	21/104	20.2%	0.26 (0.15 - 0.46)	0.16 (0.08 - 0.32)	p<0.0001
	RURAL REGIONAL	32/94	34.0%	0.54 (0.32 - 0.89)	0.09 (0.03 - 0.24)	p<0.0001
SERVICE	Metro1	93/271	34.3%	1.00	1.00	
	Regional1	26/68	38.2%	1.19 (0.68 - 2.05)	3.61 (1.7 - 7.68)	p<0.0001
	Regional2	36/67	53.7%	2.22 (1.29 - 3.82)	10.95 (3.75 - 31.99)	p=0.001

N=3 missing.

N=6 missing.

N=32 missing.

**Figure 2:**
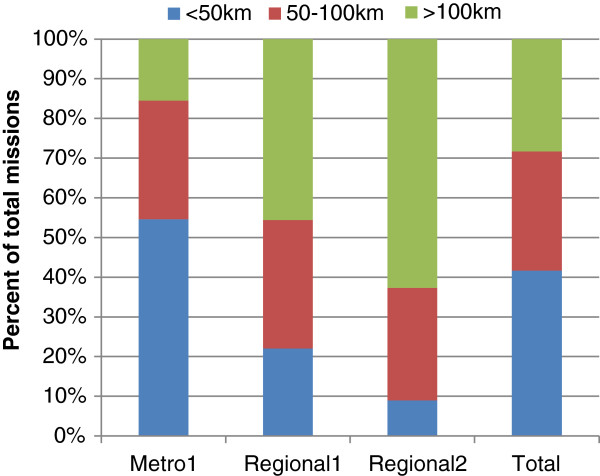
Proportion of primary scene responses within estimated distance categories to the receiving hospital stratified by service.

Figure 2 shows that the metropolitan HEMS transported the majority of their patients to a hospital within 100km of the scene (Metro1: 85%), where in contrast, the regional HEMS transported a higher proportion of patients to a hospital that was greater than 100km from the scene (Regional1: 46%; Regional2: 63% respectively). Metro1 travelled a median distance of 44km (IQR: 24-78km) to the receiving hospital whereas Regional1 and Regional2 travelled median distances of 94km (IQR: 54-131km) and 114km (IQR: 83-180km) to the receiving hospital respectively.

**Figure 3:**
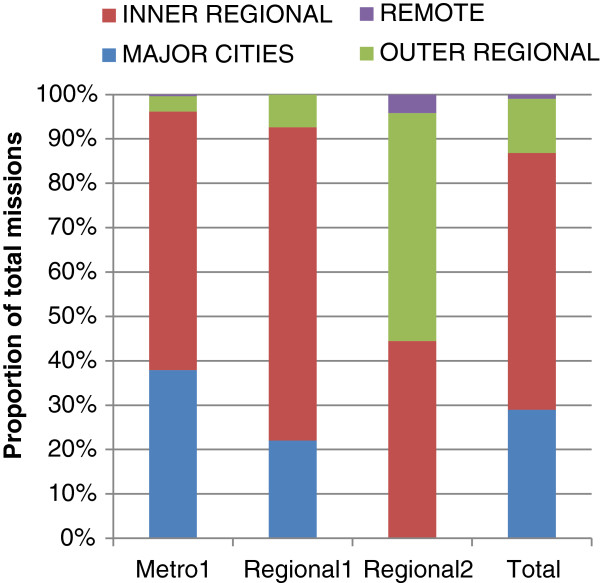
Proportion of primary scene responses within ARIA+ categories of remoteness.

Figure 3 provides an overview of the remoteness of HEMS activities. Primary scene responses for the metropolitan HEMS were predominately classified as either ‘major cities’ or ‘inner regional’. In contrast, Regional2 HEMS responded to a majority of localities classified as ‘outer regional’ or ‘inner regional’.

Table 3 provides an overview of all patients attended by HEMS (including non-emergency and winch missions; N=596). The majority of patients were male (n=434, 74%) and adults with 13% (N=78) aged over 60 (median age: 37; IQR: 20–51). In terms of primary scene responses attended by HEMS, most were trauma related (N=555, 93%) with road trauma the predominant cause of trauma-related incidents (N=259, 47%). For trauma patients, injuries to the extremities were the most common (N=238, 31%).

**Table 3:** Characteristics of patients attended by HEMS in primary scene responses

**Patient characteristics**	**N (%)**
AGE	(N=589; 7 missing)
<=16	89 (15.1%)
17-30	162 (27.5%)
31-45	147 (25%)
46-60	113 (19.2%)
61+	78 (13.2%)
SEX	(N=590; 6 missing)
MALE	434 (73.6%)
FEMALE	156 (26.4%)
INCIDENT	(N=596; 0 missing)
ROAD	259 (43.5%)
FALL	110 (18.5%)
SPORT	66 (11.1%)
OTHER	71 (11.9%)
UNKNOWN^1^	49 (8.2%)
NON-TRAUMA	41 (6.9%)
PATIENT DIAGNOSIS	
TRAUMA DX^2^	(N=555; 0 missing)
ABDOMEN	23 (3%)
BURNS	12 (1.6%)
CHEST	70 (9.2%)
EXTREMITY	238 (31.4%)
HEAD	180 (23.7%)
MULTI	115 (15.2%)
OTHER	30 (4%)
PELVIS	32 (4.2%)
SPINAL	101 (13.3%)
NON-TRAUMA DX	(N=40; 1 missing)
CARDIOVASCULAR	16 (2%)
GASTROINTESTINAL	3 (0.4%)
METABOLIC	7 (1%)
NEUROLOGIC	3 (0.4%)
OTHER	8 (1%)
RESPIRATORY	3 (0.4%)
CLINICAL CONDITION	(N=595; 1 missing)
DIED PRE ARRIVAL	9 (1.5%)
DIED POST ARRIVAL OR ENROUTE	11 (1.9%)
UNSTABLE INITIALLY AND DIDNT IMPROVE	27 (4.5%)
UNSTABLE INITIALLY AND IMPORVED	74 (12.4%)
STABLE INITIALLY AND DETERIORATED	8 (1.3%)
STABLE THROUGHOUT	466 (78.3%)
PHYSIOLOGY^3^	
GCS	(N=538; 58 missing)
3-8	57 (10.6%)
9-14	87 (16.2%)
15	394 (73.2%)
RTS	(N=486; 110 missing)
<7.84	92 (18.9%)
7.84	394 (81.1%)

‘Unknown’ incidents included trauma related incidents such as head injuries where information regarding the incident is missing.

Patients with multiple injured body regions (e.g. Pelvis/Spinal Trauma) were listed in all identified body regions and hence percentages add to greater than 100.

Patients considered ‘dead pre arrival’ and non-trauma patients excluded.

Table 3 also shows patient clinical condition as judged by the attending clinician as well as patient physiology at arrival. We found 2% of patients (N=9) were dead on arrival of the HEMS team with a further 2% (N=11) dying post-arrival or en-route to hospital. The majority of patients were considered stable at arrival (N=474, 80%), with the majority of these patients remaining stable (N=466, 98% of initially stable patients). Seventeen percent of patients (N=101) were considered unstable at arrival. For trauma patients (excluding patients who were dead on arrival), we found 73% of patients had a normal GCS score of 15 (N=394). We were also able to calculate the Revised Trauma Score for 486 patients (88% of trauma patients) with 81% of these patients recording a normal score of 7.84 (N=394).

Table 4 shows patient characteristics in relation to the clinical condition at arrival. For patients that died before or after the HEMS arrival or en-route to the hospital, we found a high proportion of non-trauma incidents (33% and 46% respectively). The majority of these non-trauma responses were classified as due to cardiovascular events (22% and 36% respectively) such as cardiac arrest.

**Table 4:** Characteristics of patients stratified by clinical condition on arrival at the scene

	**Died pre-arrival**		**Died post-arrival**		**Unstable initially and didn’t improve**		**Unstable initially and improved**		**Stable initially and deteriorated**		**Stable throughout**		**Total**	
	**N**	**% TOTAL**	**N**	**% TOTAL**	**N**	**% TOTAL**	**N**	**% TOTAL**	**N**	**% TOTAL**	**N**	**% TOTAL**	**N**	**% TOTAL**
TOTAL^1^	9		11		27		74		8		466		595	
MECHANISM OF INJURY														
FALL	3	33%	1	9%	3	11%	12	16%	0	0%	91	19.5%	110	18.5%
NON-TRAUMA	3	33%	5	46%	2	7%	8	11%	1	12.5%	21	4.5%	40	6.7%
OTHER	2	22%	1	9%	2	7%	7	10%	2	25%	57	12.2%	71	11.9%
ROAD	1	11%	3	27%	17	63%	39	53%	4	50%	195	41.8%	259	43.5%
SPORT	0	0%	0	0%	0	0%	5	7%	0	0%	61	13.1%	66	11.1%
UNKNOWN	0	0%	1	9%	3	11%	3	4%	1	12.5%	41	8.8%	49	8.2%
TRAUMA LOCATION														
ABDOMEN	0	0%	0	0%	0	0%	4	5%	0	0%	19	4.1%	23	3.9%
BURNS	0	0%	0	0%	0	0%	1	1%	1	12.5%	10	2.1%	12	2%
CHEST	0	0%	1	9%	5	19%	6	8%	2	25%	56	12%	70	11.8%
EXTREMITY	0	0%	0	0%	2	7%	21	28%	3	37.5%	212	45.5%	238	40%
FACE	0	0%	1	9%	0	0%	4	5%	0	0%	18	3.9%	23	3.9%
HEAD	1	11%	2	18%	21	78%	34	46%	1	12.5%	121	26%	180	30.3%
MULTI	1	11%	4	36%	15	56%	23	31%	1	12.5%	71	15.2%	115	19.3%
OTHER_TRAUMA	3	33%	2	18%	1	4%	7	10%	0	0%	17	3.6%	30	5%
PELVIS	0	0%	0	0%	0	0%	4	5%	3	37.5%	25	5.4%	32	5.4%
SPINAL	0	0%	0	0%	0	0%	3	4%	0	0%	98	21%	101	17%
NON-TRAUMA DX														
CARDIOVASCULAR	2	22%	4	36%	1	4%	3	4%	1	12.5%	5	1.1%	16	2.7%
GASTROINTESTINAL	0	0%	0	0%	0	0%	0	0%	0	0%	3	0.6%	3	0.5%
METABOLIC	0	0%	0	0%	0	0%	2	3%	0	0%	4	0.9%	6	1%
NEUROLOGIC	0	0%	1	9%	1	4%	0	0%	0	0%	1	0.2%	3	0.5%
OTHER	1	11%	0	0%	0	0%	1	1%	0	0%	6	1.3%	8	1.3%
RESPIRATORY	0	0%	0	0%	0	0%	2	3%	0	0%	1	0.2%	3	0.5%

N=1 missing.

## Discussion

To date, this is the most comprehensive description of both metropolitan and regional HEMS primary scene responses in NSW which includes the time taken and the proximity of missions to the receiving hospital. Our results highlight the often large distances travelled by HEMS in NSW in transport to the receiving hospital. During patient transport, HEMS in NSW often bypass the nearest designated trauma hospital as part of the local regionalised trauma care system. Patients transported by HEMS during primary scene responses were predominantly judged to be clinically stable.

Around the world, HEMS operate in many different settings including urban, rural and remote environments. We found HEMS in NSW predominantly operated in areas classified as ‘major cities’ or ‘inner regional’ although differences existed between the urban and regional based HEMS. For HEMS in regional areas, we found on average a two-fold increase in the average distance travelled relative to the trauma hospital, compared to the metropolitan HEMS. Such differences suggest variance in the benefits of HEMS in NSW which includes improving health service equity in regional areas and providing a ‘second tier’ of support in urban areas. Locations of HEMS in NSW are historically determined and given the large distances travelled by HEMS in NSW, further research is needed into the most appropriate HEMS locations relative to need.

Our results regarding distances travelled by HEMS in regional areas are consistent with previous research in NSW [18]. Compared to a meta-analysis from the US of HEMS pre-hospital care times for trauma [19], our results showed HEMS in NSW have longer times in all categories. This discrepancy may reflect several features of the local system such as the large distances travelled to the scene and the use of physicians as opposed to paramedics (which are predominantly used in US HEMS). A recent analysis from California showed over 60% of HEMS primary scene responses were within 29miles (~47km) of the receiving hospital [20]. This compared to approximately 40% of missions within the same distance in our analysis. Compared to European HEMS, which are predominantly physician staffed, our NSW transport times were also longer with HEMS transport times to the receiving hospital in the Netherlands reported as a mean of 13min [21]. This compared to a mean of 30min in our study.

Our results also showed HEMS in NSW attend a large diversity of trauma incidents including road trauma, falls, sports injuries and a small proportion of patients classified as non-trauma. The predominance of road trauma reported in our study is similar to previous findings in other jurisdictions [22-24] although the proportion of incidents was slightly less than that previously reported in NSW [18,25]. The majority of patients attended by HEMS were assessed to be clinically stable and had normal physiology, although data limitations precluded a true assessment of illness severity. Previous research has documented high over-triage rates in HEMS primary scene responses [26]. Given the expense of HEMS in NSW [12], there is scope for further research into the accuracy of current dispatch criteria in NSW to ensure HEMS are targeted to appropriate patients.

An important finding in our study was the high proportion of missions that bypassed the closest designated trauma hospital. This highlights a seldom-mentioned advantage of HEMS which incorporates the crew’s ability to exercise clinical judgment and take patients to appropriate hospitals without being restricted by road networks or travel time. In practice this includes burns and spinal trauma as well as paediatric patients being transferred to specialised hospitals where definitive specific care can be provided. HEMS also bypassed lower grade trauma hospital in order to take patients to major trauma hospitals that can provide clinical services such as interventional radiology, cardiothoracic surgery and neurosurgery. We also noted a lower probability of hospital bypass by regional HEMS, which may have reflected patients who do not require neurosurgery or cardiothoracic surgery being taking to regional trauma services, as would be appropriate. More broadly, the high proportion of HEMS bypass reflects HEMS in NSW functioning as part of a regionalised trauma care system that has been shown to reduce mortality [27,28].

Previous studies evaluating the effect of HEMS on patient mortality, have predominantly compared HEMS to a direct scene transport via ground [7,8]. In the NSW jurisdiction, our findings regarding distances travelled and the frequency of hospital bypass, highlight that HEMS are likely to replace ground transport to a regional hospital in some instances. Depending on patient acuity, this may be followed by stabilisation and subsequent transport to a major trauma hospital. Hence, when examining the economics of HEMS transports, future studies need to consider the appropriate alternative to HEMS and the full “opportunity cost” of withholding HEMS.

### Limitations

Due to inconsistent data collection we were unable to include four ‘regional’ HEMS which undertake primary scene responses in NSW in this analysis. In future this limitation will be addressed through the introduction of a state wide uniform HEMS database (Air Maestro). The HEMS that we were able to include are representative of both metropolitan and regional HEMS activities and together are responsible for approximately half of the total HEMS primary scene response activities in NSW [12]. The service excluded from our study (Metro2), operated in predominantly urban areas for the sole purpose of a clinical trial of rapid responses to head injured patients. Although by omitting this service the reported number of patients attended by HEMS in the study period with head injuries and (other factors) is likely to be underestimated, the unique nature of the Metro2 service during the study period meant the data would not be representative of traditional HEMS in the state.

Given the resource implications of using HEMS, robust service data collection is essential to investigate HEMS efficiencies and patient impact. As part of our findings, we identified several limitations in the service databases which can be addressed to improve the validity of future studies. This included omitted variables and the internal validity of collected data. In terms of omitted variables; we were unable to calculate HEMS times relative to the time of injury, however our results still provide an accurate reflection of the time taken to complete HEMS missions from activation. Data limitations also precluded the identification of patient entrapment which would have extended scene time in certain cases. Internal validity is also an issue in the data we report; as we were unable to verify data accuracy. Variables such as patient diagnosis and clinical condition rely on the opinions of multiple clinicians which may include inconsistencies. To address this issue, future database reconfiguration could consider linkage to hospital trauma registries to gain more accurate information on patient diagnosis, injury severity and outcomes.

## Conclusion

Assessing the benefit of HEMS in primary scene responses is difficult as HEMS encompasses a ‘package’ of interventions including improved access, speed and advanced clinical skills and decision making, which is known to vary between regions. Describing the characteristics of HEMS missions and patients in the local environment is an important step in understanding how HEMS benefit the health system. Our results document the time HEMS take to activate, respond to the scene, treat and transport patients as well as the proximity of HEMS operations to the receiving hospital and the clinical characteristics of patients attended. The high proportion of hospital bypass is a seldom mentioned benefit of HEMS and this finding has implications for future studies assessing the true benefit a HEMS provides. Importantly, our results highlight many areas for future research to ensure HEMS are used efficiently and appropriately.

## Abbreviations

HEMS: Helicopter Emergency Medical Service; GIS: Geographic Information Systems; GCS: Glasgow Coma Score; RTS: Revised Trauma Score.

## Competing interests

The authors declare that they have no competing interests.

## Authors’ contributions

CT conceived this study, carried out the statistical analysis and drafted the original manuscript. BL provided assistance with statistical analysis and reviewed the manuscript. EB undertook geographical mapping and reviewed the manuscript. BB, SJ and JM provided clinical and health service expertise and reviewed the manuscript. All authors read and approved the final manuscript.

## Pre-publication history

The pre-publication history for this paper can be accessed here:

http://www.biomedcentral.com/1472-6963/12/402/prepub
